# Supplementary material for: Generation of Rapid and High-Quality Serum by Recombinant Prothrombin Activator Ecarin (RAPClot™)
Source: Biomolecules. 2024 May 30;14(6):645. doi: 10.3390/biom14060645 (PMC11201583; doi:10.3390/biom14060645)
Supplement: Supplementary file 1 [file biomolecules-14-00645-s001.zip › biomolecules-2978472-supplementary.pdf]

Supplementary Table S1. RAPClot prototype tubes prepared in different formulations with or without γ-radiation were used for S2238 assay (Fig. 2A)

RAPClot	Formulation	BSA (%)	RAPClot Tube code	γ-radiation (27.8 kGy)
(0.33 mU/tube)	Gelofusine (25 µL/Tube) Lactulose (10%, W/V)	0	S-A	Ir-
				Ir+
		4	S-B	Ir-
				Ir+
		8	S-C	Ir-
				Ir+
	Hepes (25 µL/tube) Lactulose (10%, W/V)	4	S-D	Ir-
				Ir+

Supplementary Table S2. RAPClot prototype tubes prepared in six formulations with or without γ-radiation were used for S2238, plasma and blood clotting assay (**Fig. 2B,C&D; S. Fig. 4**).

RAPClot	Formulation including a stabilising sugar	BSA (%)	γ-radiation (27.8kGy)	RAPClot Tube code
0.26 mU/Tube	Gelofusine (25 µL/Tube) Lactulose (10%, W/V)	0	-	A-Ir
			+	A+Ir
		4	-	B-Ir
			+	B+Ir
		8	-	C-Ir
			+	C+Ir
	Hepes (25 µL/tube) Lactulose (10%, W/V)	4	-	D-Ir
			+	D+Ir
		8	-	E-Ir
			+	E+Ir
		12	-	F-Ir
			+	F+Ir

Supplementary Table S3. Thirty-three analytes measured in serum generated from five blood collection tubes: SST, RST, RAPClot, RAPClot+Heparin(8U/mL) and PST prepared at 0 h after collection in the second trial. The data are the mean±standard deviation (SD) of participants respectively (n=5).

		SST	BDRST	RAPClot	RAPClot +Hep	PST
Analyte	Unit	AV±SD	AV±SD	AV±SD	AV±SD	AV±SD
Na	mmol/L	136.4±0.55	136.8±1.78	136.8±1.30	136.2±1.09	136.6±1.34
K	mmol/L	4.04 ±0.33	4.02±0.32	4.04±0.21	3.96±0.32	3.92±0.32
CL	mmol/L	104.4±2.07	105.6±2.88	105.6±2.60	104.4±2.30	105.4±2.50
HCO3	mmol/L	23±2	22.6±1.94	21.6±1.51	23.4±1.67	22.8±2.28
GLU	mmol/L	5.34±0.67	5.58±0.72	5.38±0.52	5.58±0.66	5.62±0.74
UREA	mmol/L	5.38±0.98	5.38±0.97	5.46±0.89	5.1±0.91	5.36±1.05
CRE	µmol/L	77.6±13.53	80.4±11.39	75.8±14.09	79±15.38	83±10.83
URAT	mmol/L	0.32±0.07	0.32±0.07	0.32±0.07	0.32±0.07	0.32±0.07
T PROT	g/L	67.4±2.60	68.4±3.91	67.8±2.77	70.8±2.86	68.4±3.91
ALB	g/L	42.4±2,61	42.6±2.88	42±2.34	41.8±2.49	42.4±2.88
T BILI	µmol/L	16.8±3.63	15±2.74	16±3.74	16±3.74	15.2±2.95
ALP	U/L	69±12.36	68.6±12.99	69.4±13.55	68.6±11.32	68.4±12.84
GGT	U/L	14.4±5.22	13.4±6.54	14.4±3.91	16.4±5.77	13.8±5.49
ALT	U/L	21.2±8.70	21±.2±9.98	21.6±10.23	21.6±8.41	20.6±9.93
AST	U/L	18.4±3.57	18.4±4.82	18.6±6.10	18.8±4.02	18.2±3.83
LD	U/L	213.8±33.16	225.8±36.87	212.4±25.11	208.8±29.30	197.2±27.41
CK	U/L	103.8±29.73	104.4±27.89	103.6±28.44	103.2±29.48	102.6±28.22
CA	mmol/L	2.33±0.14	2.31±0.13	2.29±0.12	2.29±0.12	2.30±0.14
PHOS	mmol/L	1.05±0.18	1.07±0.18	1.02±0.17	0.99±0.17	1.06±0.19
LIPASE	U/L	31.4±7.12	32±7.87	32.2±7.32	31.8±7.72	31.4±7.92
MG	mmol/L	0.84±0.03	0.85±0.02	0.83±0.02	0.83±0.02	0.85±0.03
CHOL	mmol/L	5.44±0.81	5.36±0.54	5.34±0.54	5.26±0.61	5.36±0.58
TRIG	mmol/L	1.36±0.74	1.36±0.71	1.38±0.71	1.34±0.76	1.32±0.76
HDL	mmol/L	1.6±0.34	1.58±0.34	1.58±0.32	1.6±0.30	1.56±0.32
IRON	µmol/L	19±5.61	18.6±4.82	19.2±5.6	18.2±4.96	18.6±5.50
TRF	g/L	2.44±0.27	2.46±0.23	2.46±0.20	2.4±0.23	2.46±0.21
FERR	µg/L	78.4±44.07	74.2±38.64	76.2±42.39	74±41.71	76±43.09
CRP	mg/L	1.2±0.14	1.4±0.14	1.64±0.66	1.24±0.42	1.14±0.49
TSH	mU/L	1.44±0.82	1.48±0.86	1.44±0.86	1.48±0.87	1.48±0.83
FT3	pmol/L	5.14±0.43	4.94±0.68	5.06±0.28	4.96±0.36	4.94±0.43
FT4	pmol/L	13±1.41	13±1.22	13.8±2.04	13.6±1.14	12.8±1.92
CORT	nmol/L	239.2± 46.7	236.6±44.03	234.4±49.92	240.6±47.98	239.6±46.30
SFOL	nmol/L	35.62±10.38	35.82±11.58	36.46±12.27	35.4±11.61	35.04±12.01

Supplementary Figure S1

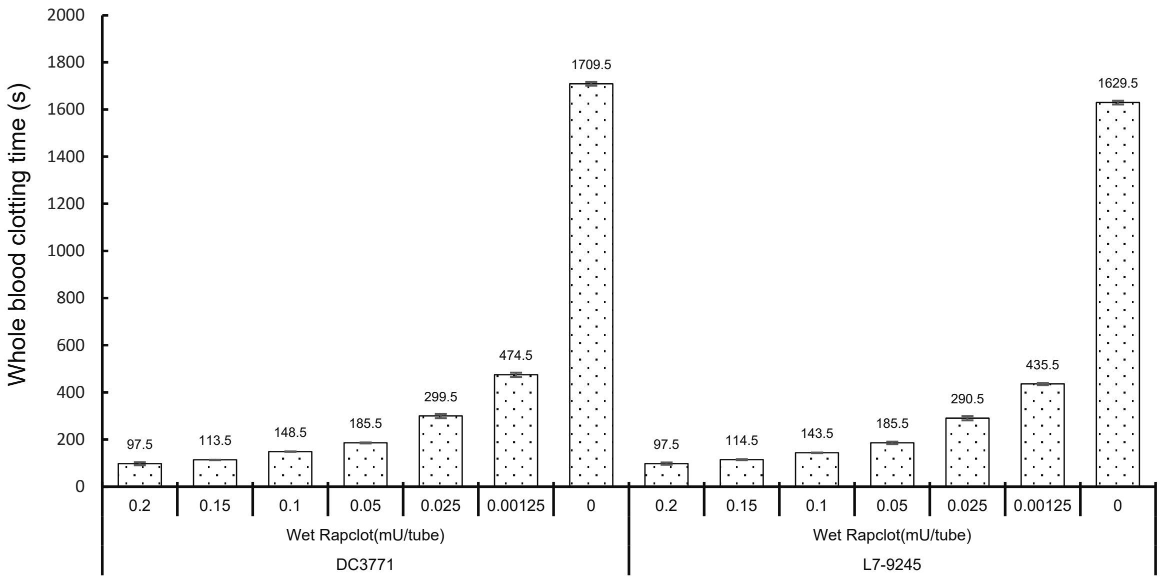

S. Figure S1. Effects of surfactants used for coating GBO white top plain blood collection tube on the activity of RAPClot in clotting wet recalcified citrated whole blood. Two surfactants, DC3771 and L7-9245 were prepared by diluting in H_2O to give a concentration of 0.5% surfactant solution (W/V). Then 20 µL of the solutions were add to the bottom surface of one GBO white top plain blood collection tube. The tubes with the added surfactant solution were vortexed for 15 s and dried by nitrogen air for 2 hr. The surfactant-coated tubes were used for blood clotting assay. RAPClot stock was used to prepare six concentrations of the RAPClot working solution in Gelofusine for clotting 3.95 mL of recalcified citrated whole blood plus 50 µl of 1 M calcium solution. The clotting procedure and clotting time record are the same as previously described (Ref).

Supplementary Figure S2

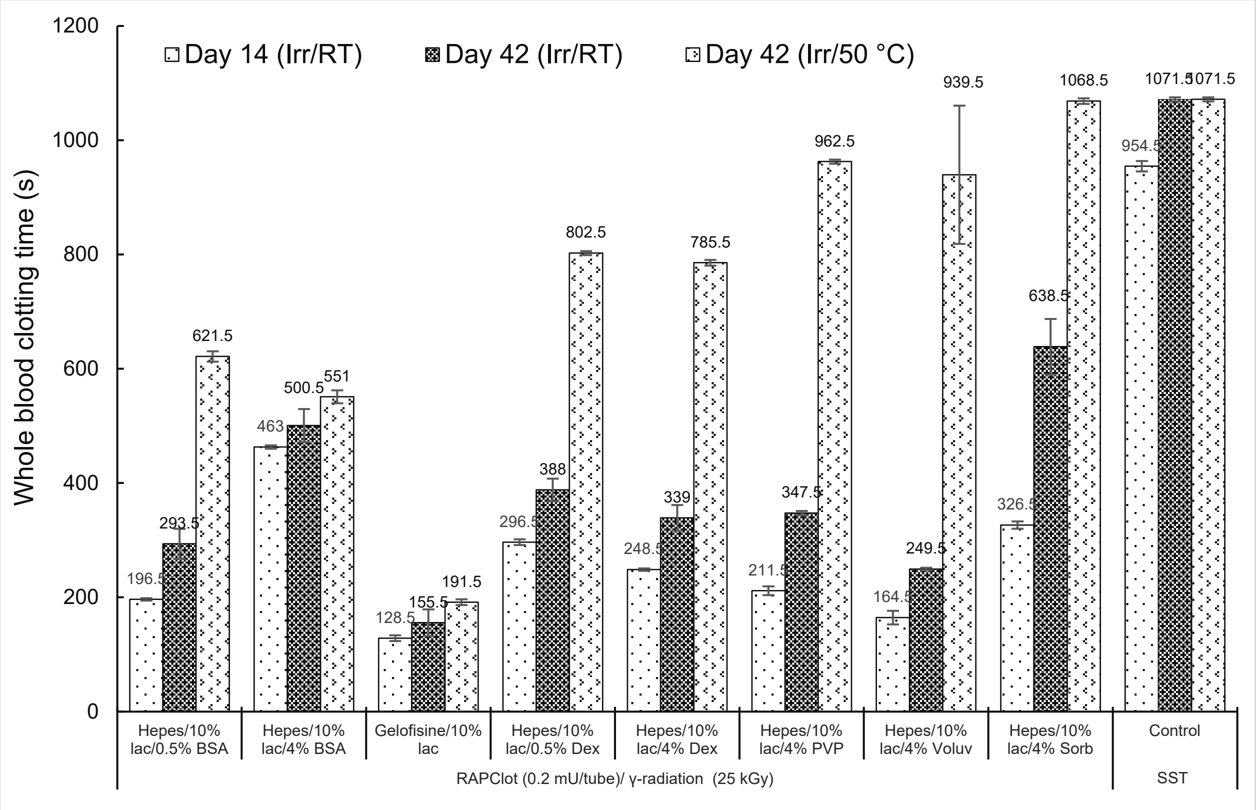

S. Fig. S2. Activity of RAPClot (0.2 mU/tube) prepared in Hepes buffer containing 10% lactulose (10% lac) plus BSA (0.5% and 4%), dextran (0.5% and 4% Dex), Polyvinylpyrrolidone (4% **PVP**), voluven (4% Voluv) and sorbitol (4% Sorb), respectively, compared with that prepared in Gelofusine containing 10% lactulose (10% lac). All the tubes were air-dried and treated by γ-radiation (25 kGy) and tested in clotting 4 mL of recalcified citrated whole blood. The RAPClot tubes were stored under both RT and 50 °C conditions up to 42 days.

Supplementary Figure S3

	Recalcified only	RAPClot tube (0.2 mU/tube)
Time(s)*	2533.7±257.8 (n=4)	89.3±3.3 (n=4)

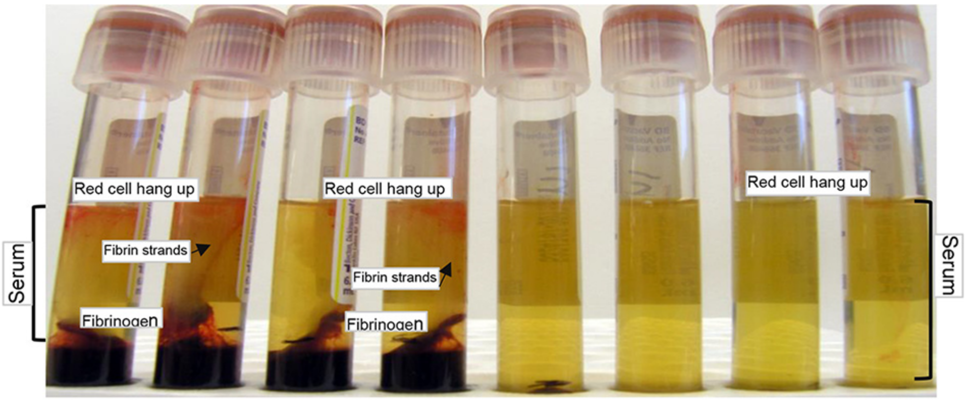

Supplementary Fig. S3. Serum quality produced by RAPClot tube compared to that by commercial BD plain rea top blood collection tube. Visual observation showed that cellular material, red blood cell hang-up, fibrin strands as well as fibrinogen were present in serum produced in all four BD commercial plain tubes while only slight red cell hang-up in one of the four tubes was observed. Note: blood clotting time(s)* was obtained in both commercial and RAPClot tubes.

Supplementary Figure S4

(A). Centrifugation scheme: 1300g/10 min

	RAPClot™ tube (2018)	BD SST no-gel tube	BD SST gel tube	BD RST-gel tube
Time(s)*	91.8±1.3 (n=5)	1857.6±91.6 (n=5)	771.2± 35.4 (n=5)	75.2± 4.3 (n=5)

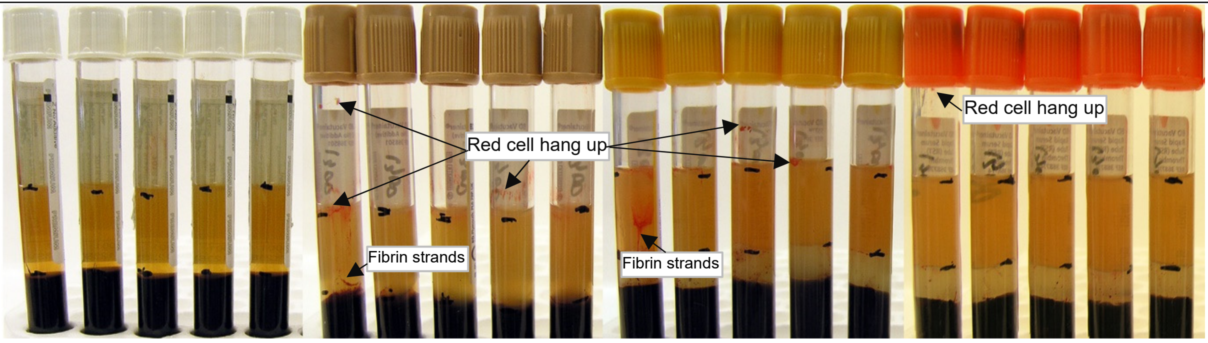

(B). Centrifugation scheme: 3000g/5 min

	RAPClot™ tube (2018)	#BD SST no-gel tube	BD SST gel tube	BD RST-gel tube
Time(s)*	92.6±1.8 (n=5)	1876.2±107 (n=5)	759.2± 36.9 (n=5)	73.0± 3.3 (n=5)

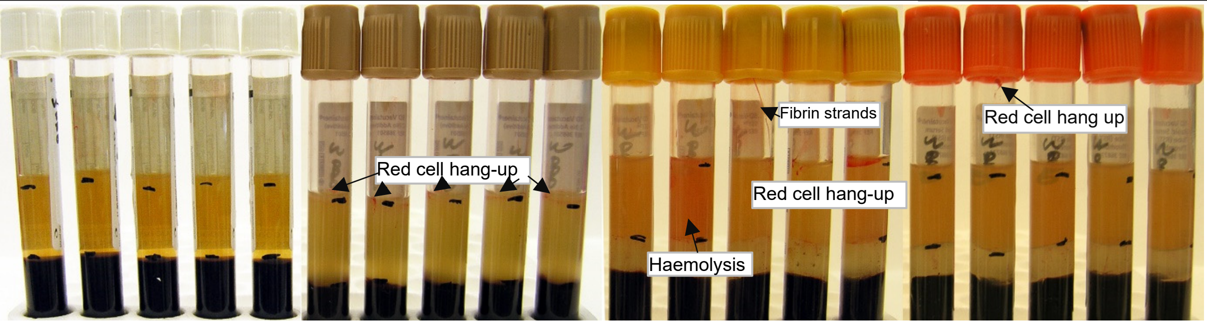

Supplementary Fig. S4. Serum quality produced by RAPClot™ tube(2018) compared to that by three commercial blood collection tubes (BD SST no gel tube, BD SST gel tube and BD RST tube). Blood clotting times (s) were recorded for all four types of the tubes. RAPClot tubes showed the clotting times significantly shorter than BD SST no gel tube, BD SST gel tube, comparable to BD RST tube. After the blood was clotted, all the tubes were centrifuged to produce serum by two centrifugation schemes: 1300g/10 min (A) and 3000g/5 min (B). Serum quality was visually examined at 18 h post-centrifugation. It was very clear that RAPClot™ tube(2018) produced the highest serum quality, without any cellular material, red blood cell hang-up, fibrin strands as well as fibrinogen observed compared to other three types of commercial tubes. # showed that latent clotting was occurred in BD SST no-gel tube at 18 h post-centrifugation.

Supplementary Figure S5

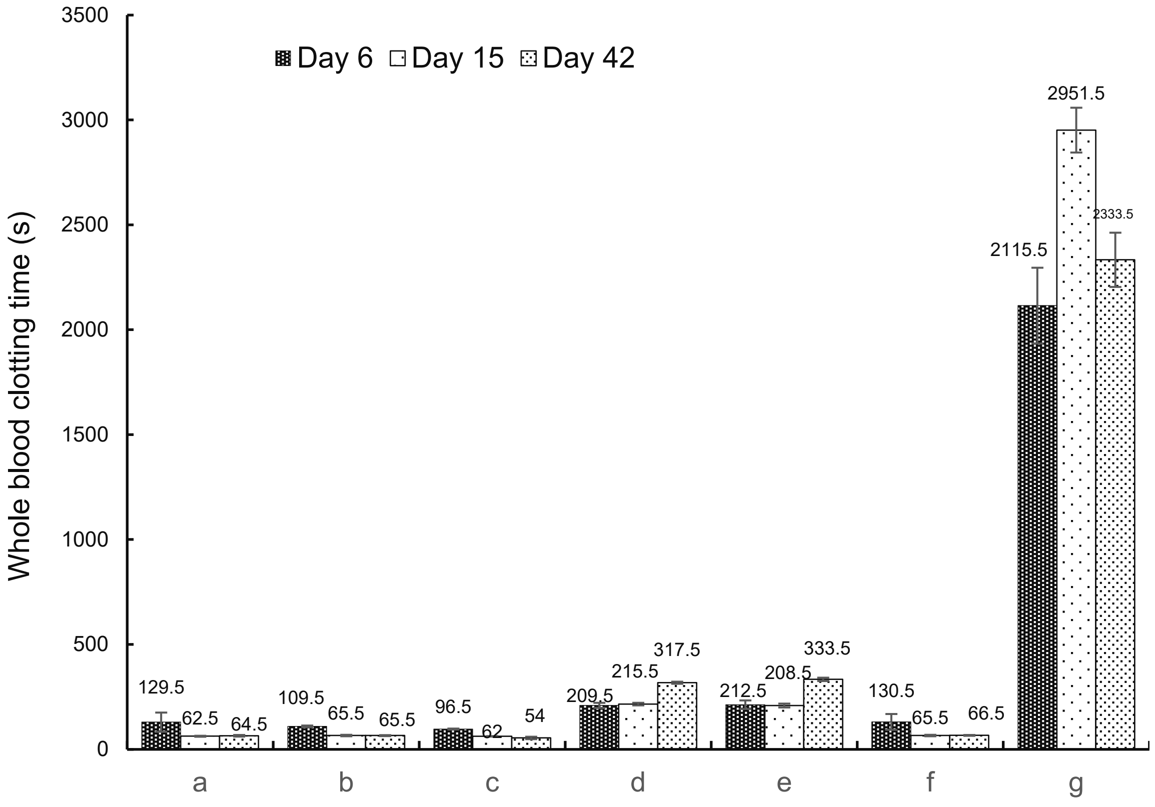

S. Figure S5. Effects of BSA as an additional component used for developing RAPClot blood clotting formulation on the activity of the wet RAPClot in clotting recalcified citrated whole blood. **(A).** RAPClot stock in a storage solution (50% Gelofusin+50% of Hepes) stored at -20 °C; **(B).** RAPClot in Gelofuine only, **(C).** RAPClot in Gelofusine+10% lactulose, **(D).** RAPClot in Gelofusine+4% lactulose+4% BSA, **(E).** RAPClot in Gelofusine+10% lactulose+4% BSA, **(F).** RAPClot in Gelofusine+10% lactulose+1% BSA and **(G).** Control (No RAPClot). RAPClot stock was used to prepare RAPClot working solution in five formations (B,C,D,E and F), 5 mL of the RAPClot working solution was prepared for each formulation, respectively, with 25 µL of RAPClot working solution containing 0.4 mU of RAPClot. The RAPClot working solutions were stored in dark at room temperature. 25 uL of RAPClot working solution (0.4 mU) was used for clotting 3.95 mL of recalcified citrated whole + 50 µL of 1 M calcium solution. The clotting procedure and clotting time record are the same as previously described (Zhao et al. 2019).

Supplementary Figure S6

(A) Plasma clotting assay

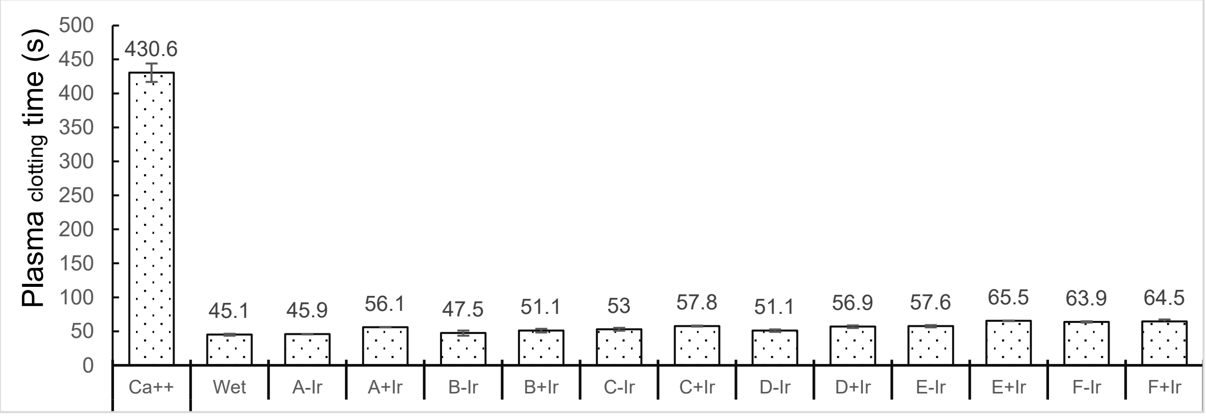

(B). Establishment of plasma clotting standard curve

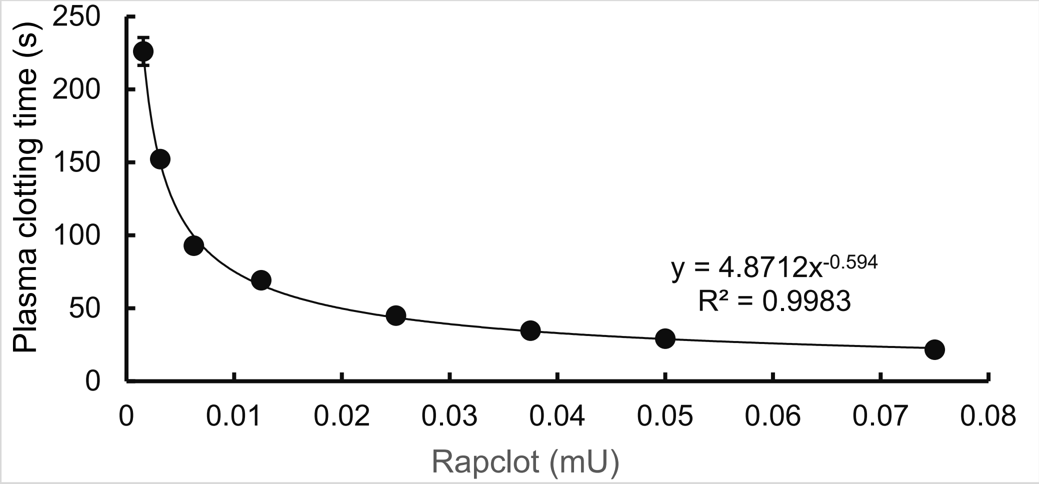

(C) Recovery of RAPClot in six formulations with or without γ-irradiation based on plasma clotting and S2238 assay

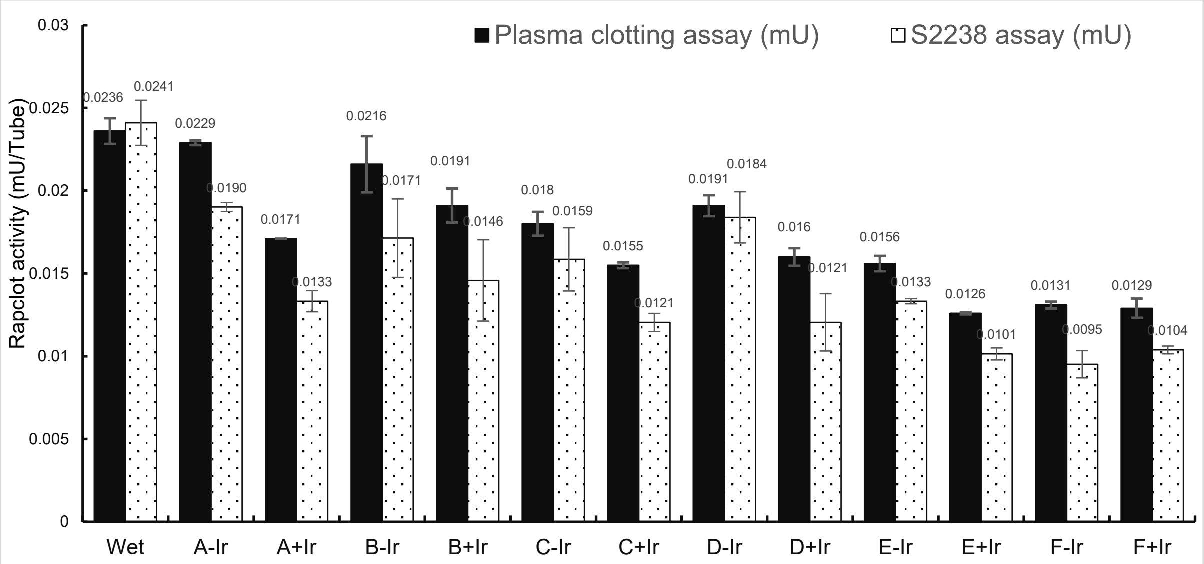

S. Fig S6. Activity of RAPClot recovered from the RAPClot prototype tubes prepared in six formulations with or without γ-radiation (27.8 kGy) according to plasma clotting and S2238 assays. As shown in Supplementary Table 2, each RAPClot prototype tube contained 0.26 mU before nitrogen dry and γ-radiation. After that 1.04 mL of Hepes buffer was added to each tube to resuspend RAPClot at day 4 post γ-radiation. Followed by 100 µL of RAPClot suspension equal to 0.025 mU was used for plasma clotting and S2238 assays. **(A).** Plasma clotting assay in a Hyland-Clotek instrument as previously described (Zhao et al. *Clin Chem Lab Med* 2019, **57**(4):483-497). 0.025 mU of RAPClot stock was used for plasma clotting as positive control. **(B)**. Plasma clotting standard curve of RAPClot stock used for estamiting the recovered RAPClot. **(C)**. Activity recovery (mU) of the RAPClot based on plasma clotting and S2238 assays in 100 µL of RAPClot suspension.

Supplementary Figure S7

(A).

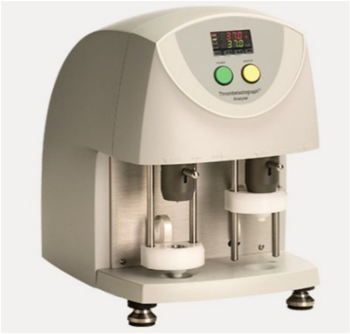

(B).

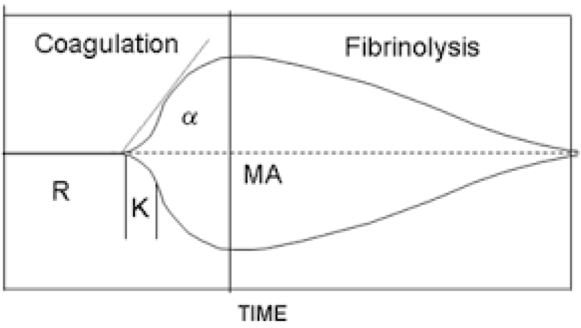

(C).

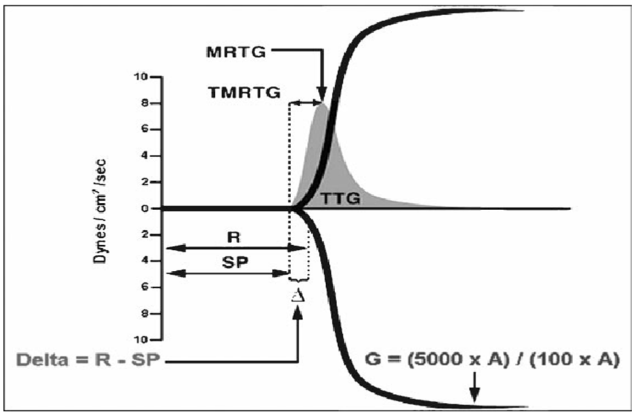

S. Fig. S7, TEG® 5000 Thrombelastograph® Hemostasis System **(A)**, Diagram of Teg including four parameters (R time, K time, α-angle and MA value) **(B)** and Diagram of thrombus velocity curve (V curve) including 1. Maximum rate of thrombus generation (MRTG), 2. Time to maximum rate of thrombus generation TMRTG and 3. Total thrombus generation (TTG) **(C).** (Haemscope Corporation, Niles, IL, USA).

Supplementary Figure S8

(A). R Time

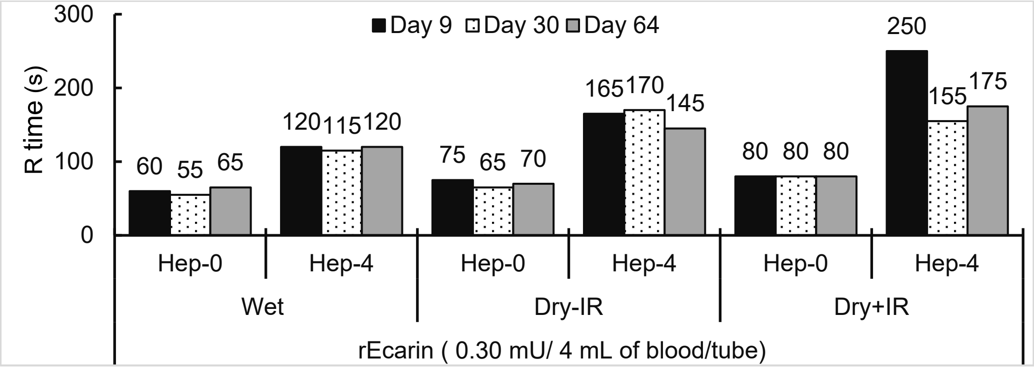

(B). K Time

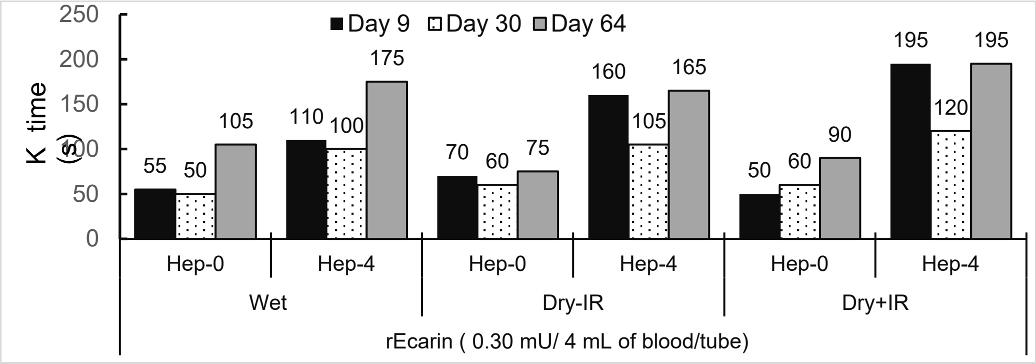

(C). Angle α

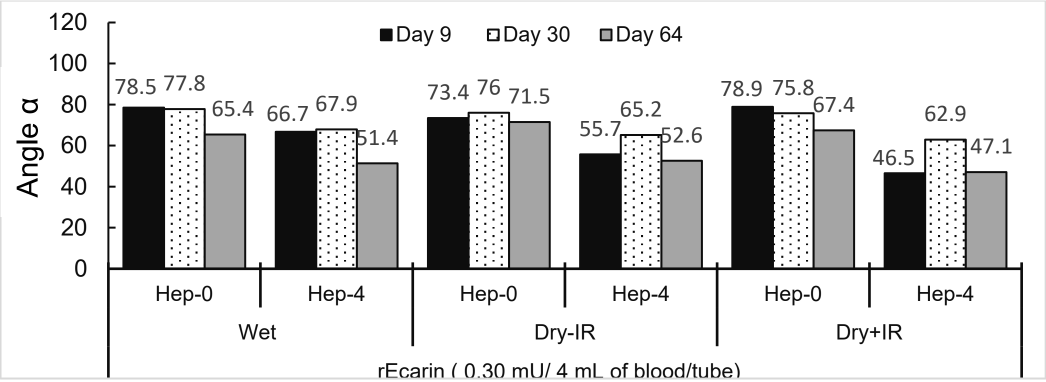

(D). MA value

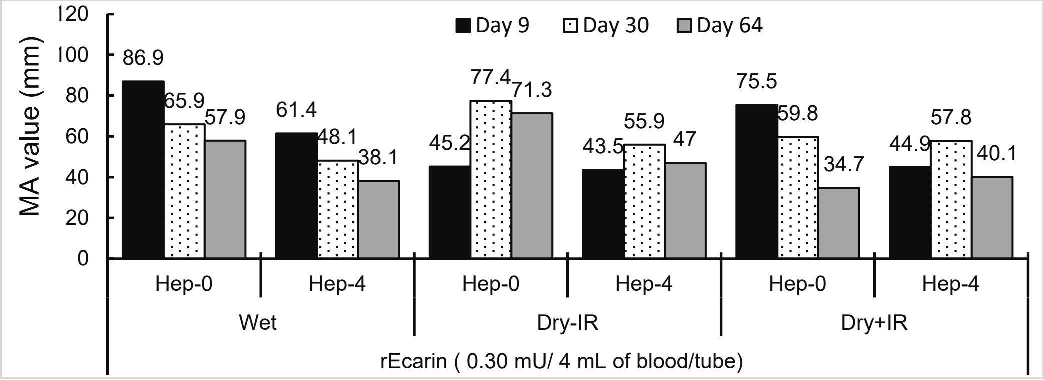

(E). MRTG

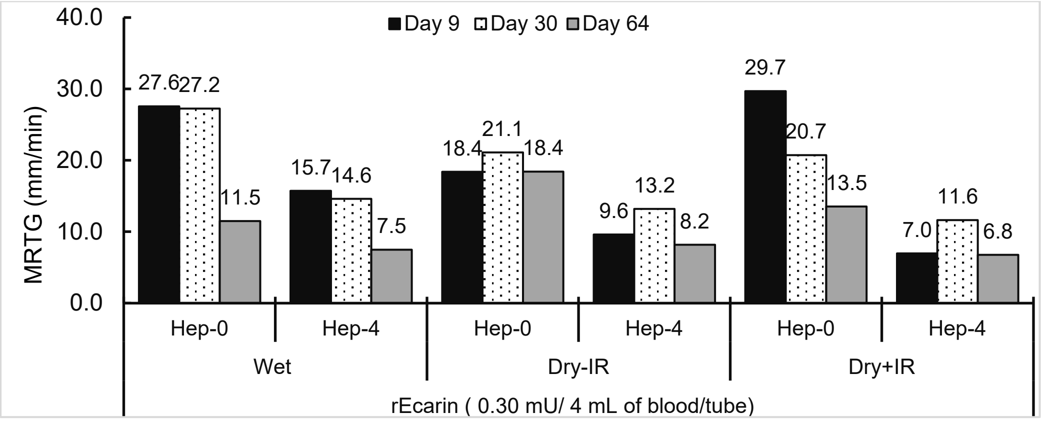

(F). TMRTG

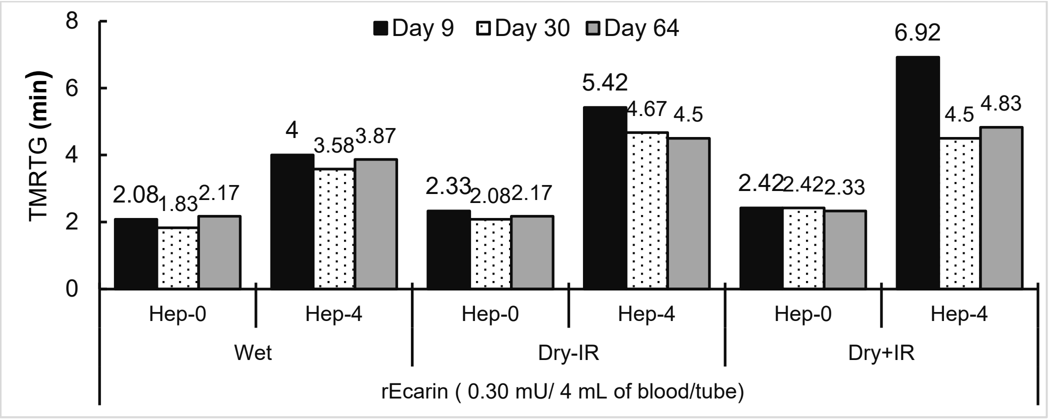

(G). TTG

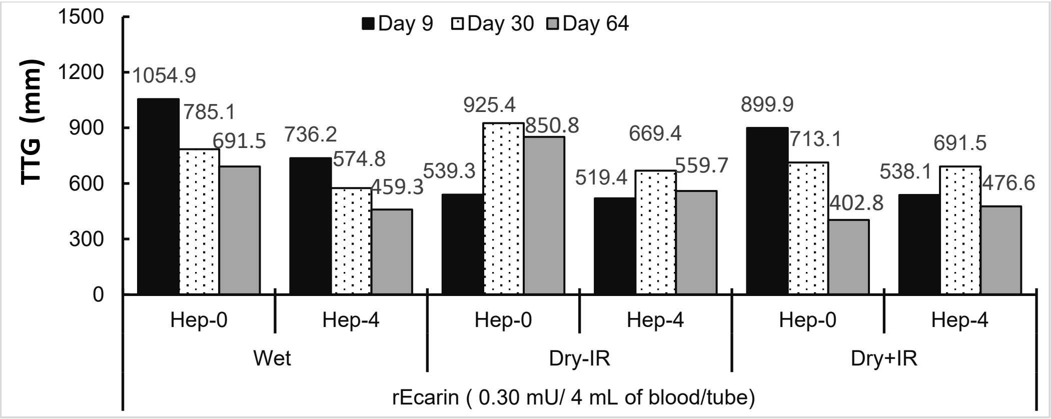

S. Fig S8. TEG assay showing the stability of RAPClot prototype-tube at dose of 0.30 mU/tube propared in one formuation with or without gamma radiation (25.7 kGy) stored for 64 days at room temperature in clotting the recalcified citrated whole bloods. **(A).** R times, **(B).** K time, **(C).** α angle values, **(D).** MA values, **(E).** *MRTG* (Maximum rate of thrombin generation), (F). T*MRTG* (Time to maximum rate of thrombus generation), **(G).** *TTG* (Total thrombus generation.

Supplementary Figure S9

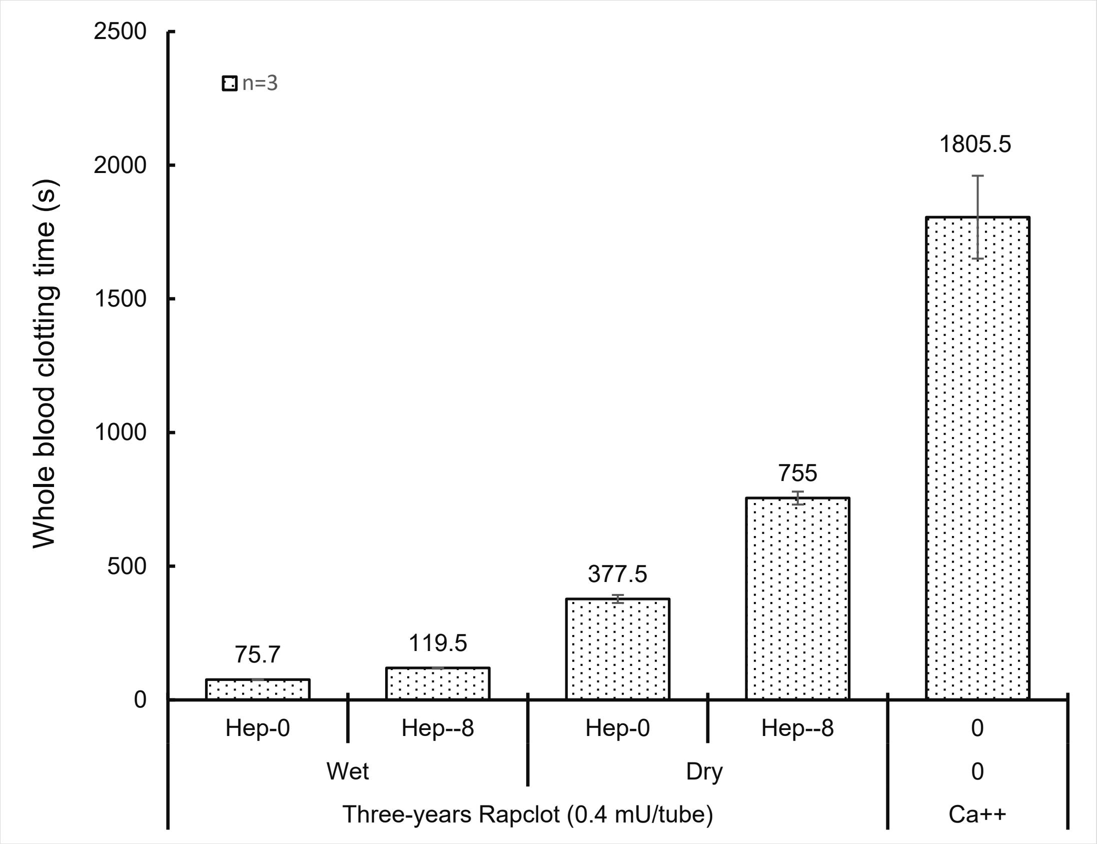

S. Fig S9. Activity of three-years RAPClot-prototype tubes in clotting 4 mL of recalcified citrated whole blood with or without heparin at 8U/mL
